# Mixed-method evaluation study of a targeted mass drug administration of long-acting anti-malarials among children aged 3 months to 15 years in the Bossangoa sub-prefecture, Ouham, Central African Republic, during the COVID-19 pandemic

**DOI:** 10.1186/s12936-024-04968-1

**Published:** 2024-05-15

**Authors:** Eve Robinson, Adelaide Ouabo, Letitia Rose, Felipe van Braak, Jorieke Vyncke, Roberto Wright, Nell Gray, Narcisse Simon Sakama, Emmanuel Joao Aboukar, Methode Mberyo Fierte, Daniel Woinzoukou, Linn Ewers, Christian Serpande, Susanne Stein, Elburg Van Boetzelaer, Odilon Auguste Kpahina, Sosthene Constant Sabe, Bhargavi Rao, Anna Kuehne

**Affiliations:** 1 Médecins Sans Frontières, Bangui, Central African Republic; 2 Médecins Sans Frontières, Bossangoa, Central African Republic; 3 Médecins Sans Frontières, London, UK; 4 Médecins Sans Frontières, Amsterdam, The Netherlands; 5 Health Region 3, Bangui, Central African Republic; 6 Ministry of Health and Population, Bangui, Central African Republic; 7 Regional University Hospital, Bossangoa, Central African Republic; 8 London School of Hygiene and Tropical Medicine, London, UK; 9 Médecins Sans Frontières, Berlin, Germany; 10 Centre for Evidence-Based Healthcare, University Hospital Dresden and Medical Faculty TUD Dresden University of Technology, Dresden, Germany

## Abstract

**Background:**

In 2020, during the COVID-19 pandemic, Médecins Sans Frontières (MSF) initiated three cycles of dihydroartemisin-piperaquine (DHA-PQ) mass drug administration (MDA) for children aged three months to 15 years within Bossangoa sub-prefecture, Central African Republic. Coverage, clinical impact, and community members perspectives were evaluated to inform the use of MDAs in humanitarian emergencies.

**Methods:**

A household survey was undertaken after the MDA focusing on participation, recent illness among eligible children, and household satisfaction. Using routine surveillance data, the reduction during the MDA period compared to the same period of preceding two years in consultations, malaria diagnoses, malaria rapid diagnostic test (RDT) positivity in three MSF community healthcare facilities (HFs), and the reduction in severe malaria admissions at the regional hospital were estimated. Twenty-seven focus groups discussions (FGDs) with community members were conducted.

**Results:**

Overall coverage based on the MDA card or verbal report was 94.3% (95% confidence interval (CI): 86.3–97.8%). Among participants of the household survey, 2.6% (95% CI 1.6–40.3%) of round 3 MDA participants experienced illness in the preceding four weeks compared to 30.6% (95% CI 22.1–40.8%) of MDA non-participants. One community HF experienced a 54.5% (95% CI 50.8–57.9) reduction in consultations, a 73.7% (95% CI 70.5–76.5) reduction in malaria diagnoses, and 42.9% (95% CI 36.0–49.0) reduction in the proportion of positive RDTs among children under five. A second community HF experienced an increase in consultations (+ 15.1% (− 23.3 to 7.5)) and stable malaria diagnoses (4.2% (3.9–11.6)). A third community HF experienced an increase in consultations (+ 41.1% (95% CI 51.2–31.8) and malaria diagnoses (+ 37.3% (95% CI 47.4–27.9)). There were a 25.2% (95% CI 2.0–42.8) reduction in hospital admissions with severe malaria among children under five from the MDA area. FGDs revealed community members perceived less illness among children because of the MDA, as well as fewer hospitalizations. Other indirect benefits such as reduced household expenditure on healthcare were also described.

**Conclusion:**

The MDA achieved high coverage and community acceptance. While some positive health impact was observed, it was resource intensive, particularly in this rural context. The priority for malaria control in humanitarian contexts should remain diagnosis and treatment. MDA may be additional tool where the context supports its implementation.

**Supplementary Information:**

The online version contains supplementary material available at 10.1186/s12936-024-04968-1.

## Background

**Fig. 1:**
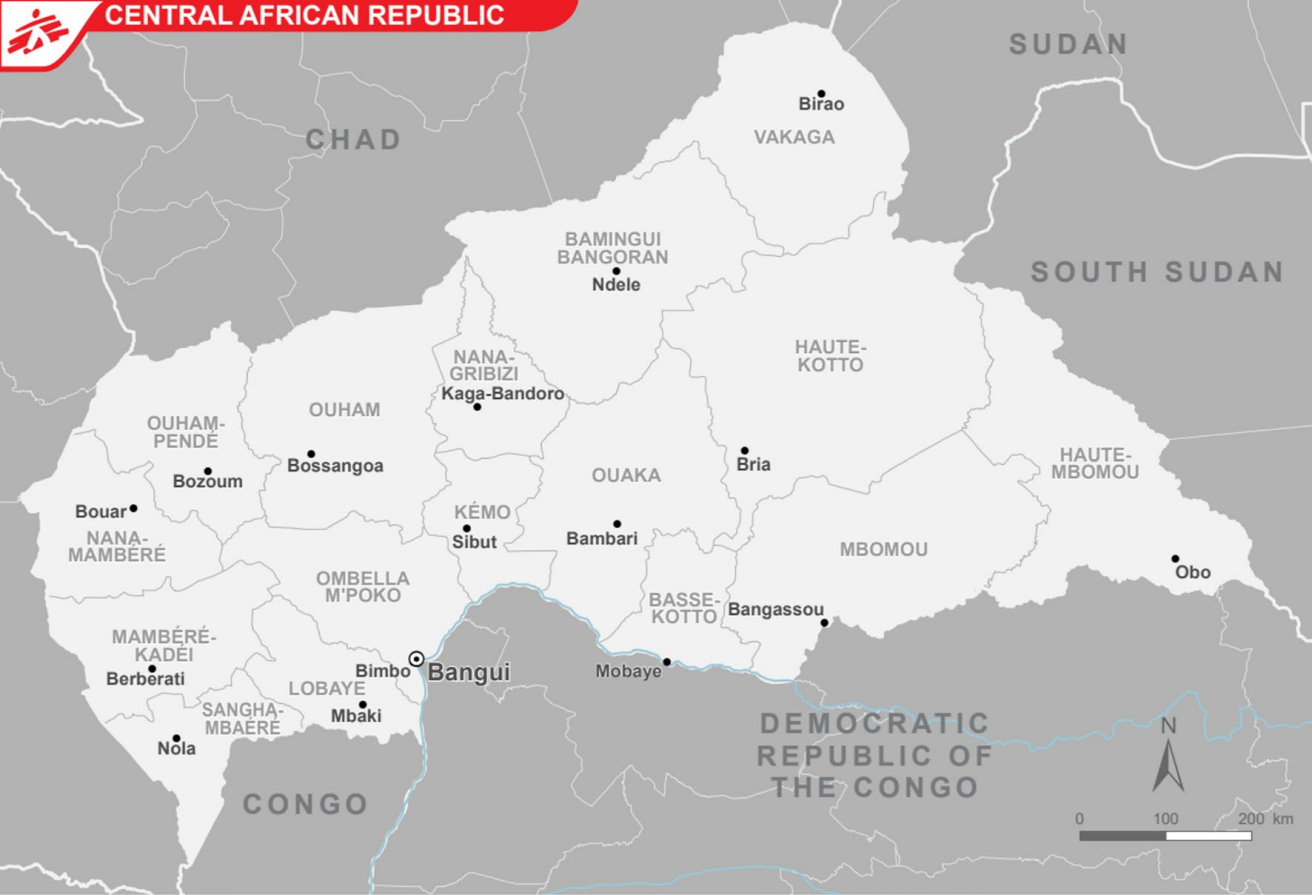
Map of the Central African Republic

The Central African Republic (CAR; Fig. 1) is ranked as the 8th most fragile state in the world [1]. Malaria is one of the principal causes of morbidity and mortality in CAR with an estimated 1.6 million cases in 2018 [2]. It is holoendemic throughout the country, with a seasonal rise typically from June to November. The main malaria parasite is *Plasmodium falciparum*.

**Fig. 2:**
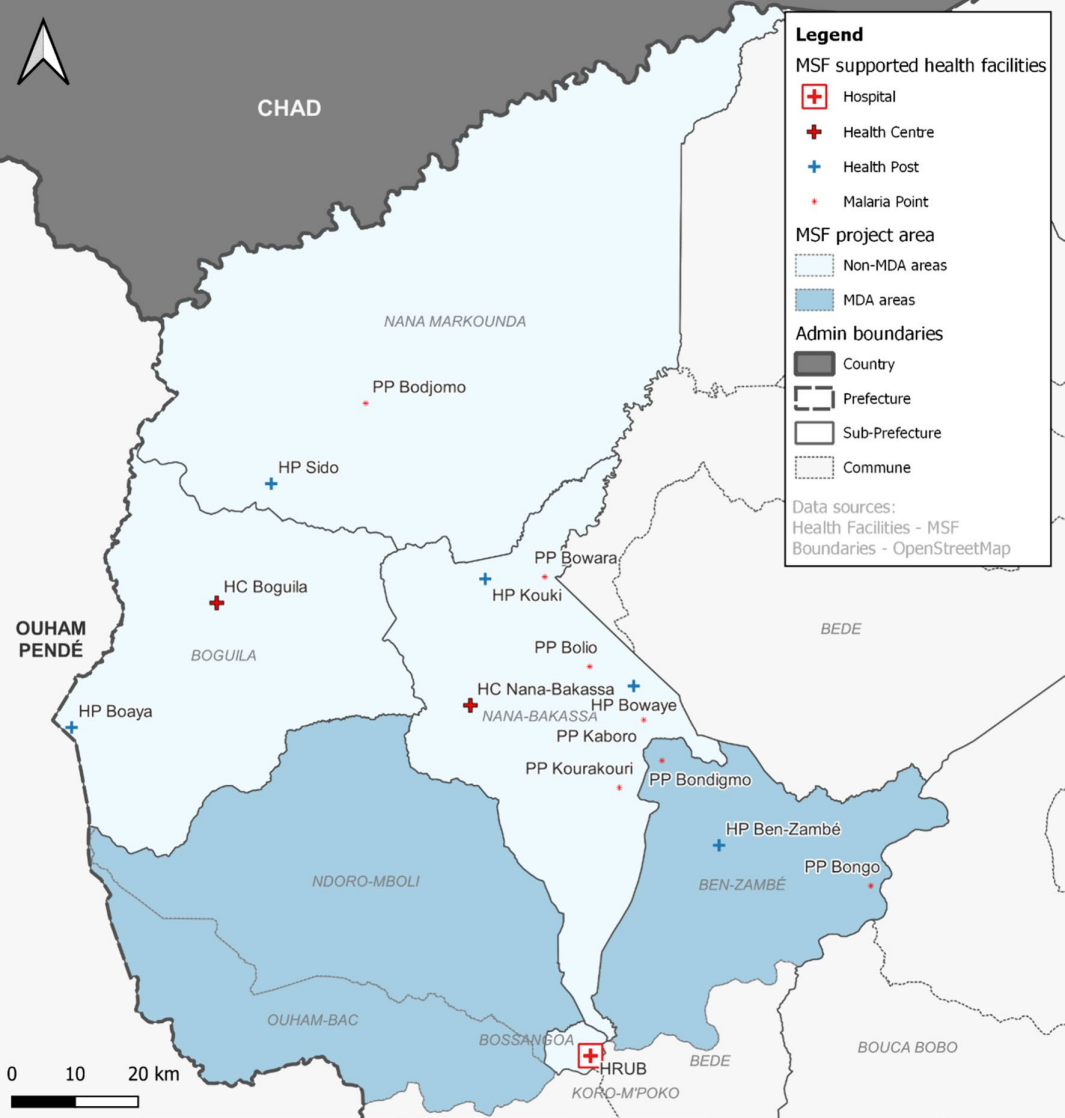
Map of the MSF project area in the sub-prefecture of Bossangoa showing the MSF supported healthcare facilities and the communes where the MDA intervention took place

In the sub-prefecture of Bossangoa, in the prefecture of Ouham, Médecins Sans Frontières (MSF) supports services in the regional university hospital (*Hôpital régional universitaire* Bossangoa—HRUB), along with several community health facilities (HFs; Fig. 2). Community HFs include health centres (HCs), health posts (HPs), and malaria points (called *points palus* (PPs)) which are community run facilities providing treatment for simple cases of malaria, diarrhoea, and respiratory tract infections. In addition to testing, treatment and referral protocols across MSF supported HFs and health promotion in the community, malaria prevention strategies employed included intermittent preventive treatment of malaria in pregnancy (IPTp), and providing insecticide-treated bed nets (ITNs) to women attending antenatal care (ANC), children with malaria and children attending a therapeutic feeding centre (TFC). Despite these preventive interventions, malaria morbidity and mortality remained high in the area. In 2019, there were: 3344 hospital admissions of children age less than 15 years with severe malaria to HRUB. In the same year there were 77,952 malaria diagnoses among children under the age of five in MSF supported community HFs in the sub-prefecture of Bossangoa, with 82% of malaria rapid diagnostic tests (RDT) performed being positive.

The World Health Organization (WHO) in its 2017 guidelines recommended mass drug administration (MDA) of anti-malarials in defined circumstances including complex emergencies or during exceptional circumstances when the health system is overwhelmed and unable to serve the affected communities [3]. This recommendation was reiterated in early 2020 as the COVID-19 pandemic propagated [4]. Given the complex humanitarian context in Bossangoa and the persistent high burden of malaria on the community, overwhelming available healthcare facilities, an MDA was planned for 2020 peak malaria season as a first step in strengthening malaria preventative measures in the region. The main aim of the MDA was not disruption of transmission but reduction in morbidity and mortality in the age-groups at highest risk of severe disease in a context of an overwhelmed health system. The rationale for this approach is the combination of a presumptive treatment effect, as well as an extended preventive effect due to the long half-life of the agents used. MSF planned an MDA for the peak malaria season of 2020. The advent of the COVID-19 pandemic and its expected impact in CAR increased its relevance [5, 6].

Starting on 17 August 2020 (week 34), MSF distributed three rounds of MDA with dihydroartemisin-piperaquine (DHA-PQ) at four-week intervals in the communes of Ouham-Bac, Ben-Zambé, and Ndoro-Mboli in the sub-prefecture of Bossangoa (Fig. 2). Due to the logistical challenges of delivering an MDA to the entire population in a conflict setting, the MDA targeted children aged 3 months to 15 years only, estimated to represent more than 50% of the population [7] including those most of risk of severe disease and death.

Two different delivery strategies were used. In both strategies, local community members (termed locally as *Relais Communautaires* or ReCos) were recruited and trained by the MSF team. Then, for larger villages—an MSF team of clinical, health promotion, administration and logistical staff went to a village on day 1 of each MDA round. They then worked with the ReCos to register eligible children and administer the first dose of the three-day course. For smaller or harder to access villages—through a partnership with the local association of motorcycle drivers (‘moto drivers’), a team of moto drivers were also trained. On day 1 they travelled to the village, bringing all the medicines and supplies. In the village they worked with the local ReCos to register the children and administer the first dose. In both delivery strategies, the doses for day 2 and day 3 were then distributed by the ReCos. During planning and implementation phases of the MDA, the MDA team undertook regular community engagement activities including meetings with community leaders and focus group discussions with community members. In 2023, the WHO updated the guidelines for malaria including chemoprevention and pointed to the lack of studies and low evidence available with regards to MDAs in humanitarian emergency settings [8]. An evaluation of the MDA in Bossangoa was conducted with the aim of contributing to the body of evidence on the effectiveness of MDAs in humanitarian emergencies. This paper focuses on the evaluation’s principal objectives, which were (1) to estimate the coverage of the MDA and (2) to estimate its clinical impact on malaria morbidity and mortality. The community member’s perception and satisfaction with the MDA will also be described.

## Methods

The evaluation included multiple components as summarized in Table 1. These included: (1) a household survey, (2) an analysis of MDA administrative data collected during the distribution, (3) an analysis of routinely collected surveillance data from MSF-supported HFs in the MDA area and HRUB, (4) analysis of enhanced surveillance data collected during the MDA from one MSF supported HP, and (5) focus group discussions (FGDs) with community members. As several of the evaluation components shared objectives, findings from different evaluation components were triangulated to give a more complete understanding. In this paper methods and findings by each objective will be presented.

**Table 1:** Overview of evaluation components and the objectives(s) each addressed

Objectives of the MDA evaluation	1. Household survey	2. Analysis of MDA data	3. Analysis of routine surveillance data	4. Analysis of enhanced surveillance data	5. Focus group discussions
Primary objectives					
Estimate coverage of the intervention	√	√			
Estimate the clinical impact on malaria morbidity and mortality	(√) morbidity only		√	√	√
Secondary objectives					
To estimate coverage of each round	√	√			
To determine differences in coverage by demographic factors	√				
To assess the community member’s perception and acceptability, including reasons for non-participation and non-adherence	√				√

### Coverage

Coverage was estimated through both a population-based household survey and administrative data collected during the MDA by the MSF team.

#### Household survey-coverage estimate-method

The household survey was conducted in the MDA area between 24 November and 9 December 2020; between 25 and 43 days since the last MDA round. The study population was children aged 3 months to 15 years (the MDA target age group).

Two-stage cluster sampling was used as per a previously described methodology [7]. Briefly, clusters were distributed amongst the three communes of the MDA area proportional to population size using estimates for 2019 from the Central African Institute for Statistics and Social and Economic Studies (Institut Centrafricain des Statistiques et des Etudes Economiques et Sociales*;* ICASEES) [9]. Cluster starting points were then randomly selected using a geographical dataset of all building footprints in CAR [10]. From the cluster starting point, subsequent households were selected in a sequential manner by selecting the next closest building to the right until the target number of eligible households were included. Households with members aged 3 months to 15 years were considered eligible.

Using ENA software for SMART 2011 [11], a required sample size of 336 households and 1046 children was estimated based on an expected MDA coverage of 50%, precision of ± 5%, design effect (DEFF) of 2.5, average household size of 7 persons [8], 52% of the population being in the target age-group (8), and a non-response rate of 5%. After an upward adjustment of 10% to account for potentially inaccessible clusters, the aim was to sample 38 clusters of 10 households each.

In eligible households, structured interviews (Additional file 1) were conducted with the head of household or a designate using KoBoToolbox on smartphones [12]. For each child aged between 3 months and 15 years, a series of questions on participation in the MDA were asked, including reasons for non-participation. Participation was noted as by verbal report or by MDA card (a card marking the administration of each MDA dose which was to be completed by the MDA distributors and given to the child’s caregiver) if it was available.

To account for infants who may not have been 3 months at the time of the MDA distribution, the estimate of coverage was restricted to children 6 months of age or older for round 1, to children 5 months of age or older for round 2, and to children 4 months or older for round 3. The estimate of overall coverage, i.e., participation in all 3 rounds, was restricted to children 6 months of age or older. Estimates of coverage are presented as proportions with 95% Confidence Intervals (CIs). Differences in proportions were measured using Pearson χ2 test and present a p-value (p). Quantitative data analysis was undertaken using Stata version 15.1 [13].

#### Analysis of MDA administrative data-coverage estimate -method

For a second estimate of coverage, data on the number of participating children collected during the MDA was used. As recent census data was not available, two different estimates of the total population were used as the denominator. The first estimate used population data provided by the communities during the preparation phase of the MDA. The method used by each village to determine this estimate was not known. The second estimate used population estimates by commune from ICASEES which were based on a 2003 census adjusted for population growth [9]. For both estimates the target population was calculated based on an estimate that 52% of the population were in the target age group aged 3 months to 15 years, as was found in a population survey in another prefecture of CAR earlier in 2020 [7].

### Clinical impact

To estimate the clinical impact of the MDA on malaria morbidity, questions on recent illness were included during the household survey, routine surveillance data from MSF supported HFs serving the MDA area was analysed, and additional data on MDA participation and RDT results at one HF in the MDA area were collected.

#### Household survey-illness in the preceding 4 weeks among children participating in the household survey - method

During the household survey questions were asked about illness experienced by each child in the household within the targeted age group in the preceding 4 weeks. For children who had been ill, additional questions were asked on symptoms and health-seeking behaviour. The proportion of children who were ill among those who had participated in the MDA to those who had not were compared.

#### Analysis of routine surveillance data-admission at HRUB and community consultations in MSF supported community HFs - method

Within the MDA intervention area, MSF supported a HP in Ben-Zambé village, and two PPs in Bondigmo and Bongo, all in the commune of Ben-Zambé. There were no MSF supported HFs in the two other communes of the MDA area. HRUB, although not in the MDA area, was the closest secondary care centre to which severe cases of malaria in the MDA area would be referred.

Data for week 1 2018 to week 53 2020 were extracted for these four HFs (HRUB, Ben Zambé HP, Bondigmo PP and Bongo PP) from MSF’s health information system (HIS). For the three community HFs in the commune of Ben Zambé weekly aggregate data was extracted on the number of consultations, malaria diagnoses, and RDTS performed. Consultations include all patients presenting to community HFs for assessment; malaria diagnoses include patients with a clinical diagnosis and positive RDT. Aggregate data was available for those aged under five years of age and five years and over. For HRUB, individual level data was extracted on age, area of residence, principal diagnosis, secondary diagnosis, and outcome for admitted children aged up to 15 years.

Using a Poisson regression model, the percentage change in malaria related indicators during the MDA period compared to the same period of 2018 and 2019 overall and by age groups (under 5 years, 5 years and older) were estimated. For the community HFs, the weekly number of consultations, malaria diagnoses and the percentage of RDTs that tested positive were compared. For HRUB, the percentage change in the number of severe malaria admissions, any malaria admission, and all admissions were estimated. The HIS contains one coded variable for the primary diagnosis of admissions. Admissions with severe malaria were identified using this primary diagnosis field and the diagnostic code for severe malaria. The HIS also contains a free-text field for any secondary diagnoses. For cases with a primary diagnostic code other than severe malaria, this free-text field was searched for text related to malaria to identify admissions with a secondary diagnosis of malaria. Any malaria was defined as a patient with a primary diagnostic code corresponding with severe malaria or a secondary diagnosis of malaria. A comparison was also made between the number of in-hospital deaths among all admissions and admissions due to severe malaria among children resident in the MDA area during the MDA period compared to previous years and compared to children resident outside of the MDA area for 2020.

The MDA commenced in week 34, starting first in the commune of Ouham-Bac, followed by Ndoro-Mboli, and then Ben-Zambé. For the analysis of hospital data, the MDA period was considered to be from weeks 34–48. For the analysis of community HFs, the MDA period was considered to be from weeks 37–48, as the distribution commenced in the commune of Ben-Zambé on the Friday of week 36, and the MDA could not affect consultations in the three community HFs here prior to week 37.

### Enhanced surveillance during the MDA at Ben Zambé HP-RDT positivity by MDA participation -  method

During the MDA, additional enhanced surveillance data at Ben-Zambé HP were also collected. Weekly aggregated data on MDA participation in the preceding four weeks and RDT outcome were collected for all consultations aged 3 months to 15 years. The odds ratio of a positive RDT among those that did not participate compared to those that did was estimated.

#### Household survey-potential adverse events - method

The household survey asked if in the week following round 3, the child experienced any illness. Please note that any illness may or may not have been an adverse event (AE) related to the MDA. It was not within the competence of the survey team to determine this.

### Perceptions and experiences of the community members

To understand the community member’s experience of the MDA FGDs were undertaken, and through household level questions were included as part of the household survey.

Led by a team of Central African anthropologists, 27 FGDs in total were undertaken—three in each commune after each MDA round. Villages were purposively selected considering inclusion of larger and smaller villages; villages with high and low coverage (estimated from MDA programmatic data), villages reporting any specific issues/concerns during the MDA, and feasibility and security. Within villages, participants were selected and recruited through ReCos in collaboration with local leaders. Participants included caregivers, local authority figures and community leaders such as village chiefs, teachers, and women's representatives, and health workers including the managers of HFs and ReCos. The composition of each FGD was mixed. The original aim was to include six to eight participants per FGD, however in reality groups ranged from 12 to 18 participants as some additional people from the villages who were included to assist with translation also participated themselves.

FGDs were conducted in Sango (the national language) where possible, with translation to local dialect by community members when required. A topic guide (Additional file 2) was used, which outlined the key themes for the discussion including: reasons for participating; experiences throughout the MDA; difficulties encountered; satisfaction with the MDA; and suggestions for future rounds/MDAs. FGDs were digitally audio-recorded and transcribed into French for analysis.

In addition to the FGDs, the household survey also included open-ended questions on satisfaction with the MDA and willingness to participate in future MDAs. Both the FGD transcripts and the household survey responses were coded in Excel using a content analysis approach to identify themes and patterns from the data [14, 15]. The identified themes will be described, including a selection of illustrative quotations.

## Results

### Coverage

#### Household survey-coverage estimate - results

The household survey reached 37 of the targeted 38 clusters. In these clusters, 474 households were visited, of which 91 (19.2%) were still absent after a second visit. Of the 383 households that were present, 366 households had at least one eligible child living in the household. Data was available for 1207 children aged between 3 months and 15 years on the day of the survey, 1189 of whom were aged 6 months or over and therefore eligible for participation in all 3 rounds of the MDA.

Among the 1207 who participated in the household survey, 1.9% (95% CI 1.1–3.1%) did not participate in any round of the MDA; 6.8% (95% CI 3.6–13.0%) participated in 2 rounds; and 93.1% (95% CI 0.85.8–96.74%) participated in 3 rounds.

When restricted to children aged 6 months who were eligible for participation in all 3 rounds, the overall coverage (i.e., participation in all 3 rounds) was 94.3% (n = 1128/N = 1189; 95% CI 86.3–97.8%) including both MDA registration card and/or verbal only reporting (Table 2). Only 5.6% (n = 58; 95% CI 2.7–11.0%) of children had an MDA registration card available to document participation in all three rounds.

**Table 2:** Coverage of all three rounds of the MDA by card or by MDA registration card/verbal only report among children aged 6 months of age to 15 years (N = 1189), MDA coverage survey, Ouham CAR, 2020

	Card only^a^			Verbal report only (card not available)^b^	Card and/or verbal report^c^	
	N	% (95% CI)	n	% (95% CI)	n	% (95% CI)
Total participation	58	5.6 (2.7–11.0)	512	44.8 (34.9–54.8)	1128	94.3 (86.3–97.8)
By commune						
Ouham-Bac	28	11.4 (4.8–24.8)	128	44.6 (34.9–54.7)	216	88.2 (56.7–97.7)
Ndoro-Mboli	30	5.6 (1.9–14.9)	289	44.9 (31.3–59.2)	521	96.5 (93.9–98.0)
Ben-Zambé	0	0	213	42.1 (23.9–62.8)	391	96.8 (92.9–98.6)
By age group						
< 1	3	7.4 (2.5–20.1)	2	34.6 (20.2–52.6)	41	91.8 (74.5–97.7)
1–4 years	18	4.7 (2.0–10.5)	23	43.2 (33.7–53.2)	403	92.6 (81.8–97.2)
5–9 years	24	6.4 (2.9–13.5)	13	47.9 (37.0–56.3)	417	95.6 (87.1–98.6)
10–15 years	13	5.4 (2.7–10.8)	9	43.2 (31.0–56.3)	267	95.6 (89.1–98.3)
By sex						
Female	20	4.0 (1.8–8.6)	249	43.9 (33.3–55.1)	553	94.6 (88.0–97.6)
Male	38	7.1 (3.5–13.8)	263	45.3 (35.3–55.6)	575	94.1 (84.2–98.0)

^a^An MDA registration card indicating participation was available for all 3 rounds

^b^Participation in all 3 rounds was indicated by verbal report only

^c^Participation in all 3 rounds was indicated by either card and/ or verbal report

Coverage (by MDA registration card or verbal only report) for each round varied from 95.1% (95% CI 86.5–98.3%) in round 3 to 98.4% (95% CI 97.1–99.2%) in round 2 (Table 3). No statistically significant difference between communes, age, or sex was identified in overall coverage, or coverage by round.

**Table 3:** Estimate of MDA coverage by round based on the household survey and administrative data, MDA, Ouham, CAR, 2020

Round and commune	Estimate of coverage from the household survey	Estimates of coverage using MDA administrative data				
		Number of children participating	Estimate A: using community provided population estimates		Estimate B: using ICASEES population estimates	
			Target population	Coverage (%)	Target population	Coverage (%)
Round 1	98.1% (96.6–98.9)	43,537	62,039	70.2	51,167	85.1
Round 2	98.4% (97.1–99.2)	44,594	62,039	71.9	51,167	87.2
Round 3	95.1% (86.5–98.3)	45,986	62,039	74.1	51,167	89.9
All 3 rounds	94.3% (86.3–97.8)	134,117	62,039	72.1	51,167	87.4

#### Analysis of MDA administrative data-coverage estimate - results

Using the data on the number of children registered during the MDA, the overall coverage for all three rounds was 72.1%, and increased from 70% for round 1, to 72% for round 2 and 74% for round 3 (Table 3) when using the community provided population estimates. Using ICASEES population estimates the overall coverage for all three rounds was 87.4%, increasing from 85.1% for round 1, to 87.2% for round 2 and 89.9% for round 3.

#### Reasons for non participation

Reasons for non-particpation From the household-survey, the most frequent reason for not participating across all three rounds was that the child was absent from the village on the distribution day (Table 4). In one cluster, no children participated in round 3. They reported that the ReCo did not come to the village and asked them to go to the village where he resided instead, which was too far for them to travel.

**Table 4:** Reasons for non-participation by MDA round, MDA coverage survey, Ouham CAR, 2020

Reason	Round 1	Round 2	Round 3
Child absent on day of distribution	15	10	22
Distance to distribution point too great			15
Child too young (as reported by the household)	3	3	10
Child ill	3		
Medicines not available	2	2	
Child refused to take MDA medicine			1
Child not resident in village during round		2	4
Team would not administer to child			1
Unknown		1	1

### Clinical impact

#### Household survey-illness in the preceding 4 weeks among children participating in the household survey-results

Among children present on the day of the survey, the overall proportion reported as being ill in the preceding four weeks was 4.1% (47/1202; 95% CI 2.5–6.6%). Among children who participated in MDA round 3 it was 2.6% (29/1 144; 95% CI 1.6–4 0.3%). While among children who did not participate at all it was 30.6% (n = 18/58; 95% CI 22.1–40.8%). The incidence of gastrointestinal symptoms, respiratory symptoms, fever, headache, rash, musculoskeletal symptoms/arthralgia, and shivers was higher among those who did not participate in round 3 compared to those who did (see Additional file 3 for symptoms reported).

Twelve children (12/1194; 1.1%; 95% CI 0.4–2.8%) were reported as having malaria in the preceding four weeks. However, the malaria diagnosis was by a healthcare worker in only three children, and by a test for only one. The proportion of children reported as having malaria was higher among those who did not participate in MDA round 3 (7/52; 13.7%; 95% CI 8.5–21.3%) compared to those who did (5/1142: 0.5%; 95% CI 0.1–1.3%).

The higher proportion of illness in the preceding four weeks among those who did not participate in round 3 persisted when the analysis was stratified by bed net use by the child the previous night. No deaths among children from the household survey were identified.

#### Enhanced surveillance during the MDA at Ben Zambé HP-RDT positivity by MDA participation-results

Among children aged from 3 months to 15 years presenting to Ben-Zambé HP between weeks 38 to 46 2020, 93.3% had participated in the MDA in the previous 28 days, ranging from 86.5% to 100% by week. Among those who had participated in the MDA, the proportion with a positive RDT was 4.3% (17/393), ranging weekly from 0.0% to 11.8%. Among those who had not participated in the MDA, the proportion with a positive RDT was 78.6% (22/28), ranging weekly from 40.0% to 100%. Over the entire period, the odds of having a positive RDT among those who had not participated in the MDA compared to those who had was 18.2 (95% CI 8.1–40.7).

#### Analysis of routine surveillance data-community consultations in MSF supported community HFs-results

**Fig. 3:**
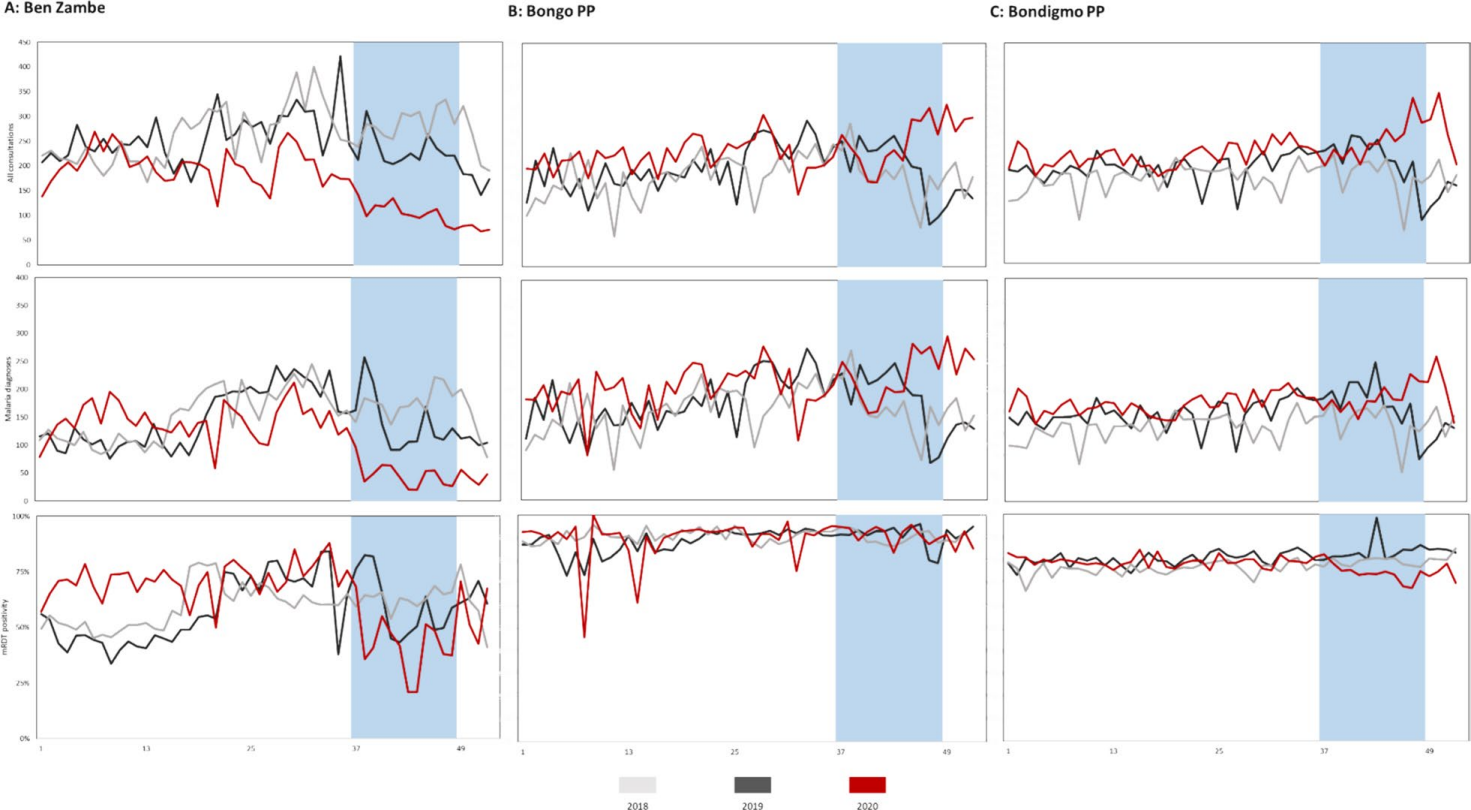
All consultations and malaria diagnoses at Ben Zambé HP, Bongo PP, and Bondigmo PP, week 1 2018 to week 53 2020

Up to the start of the MDA period in 2020, consultations, malaria diagnoses and RDT positivity in all three community HFs followed a similar pattern to previous years (Fig. 3).

**Fig. 4:**
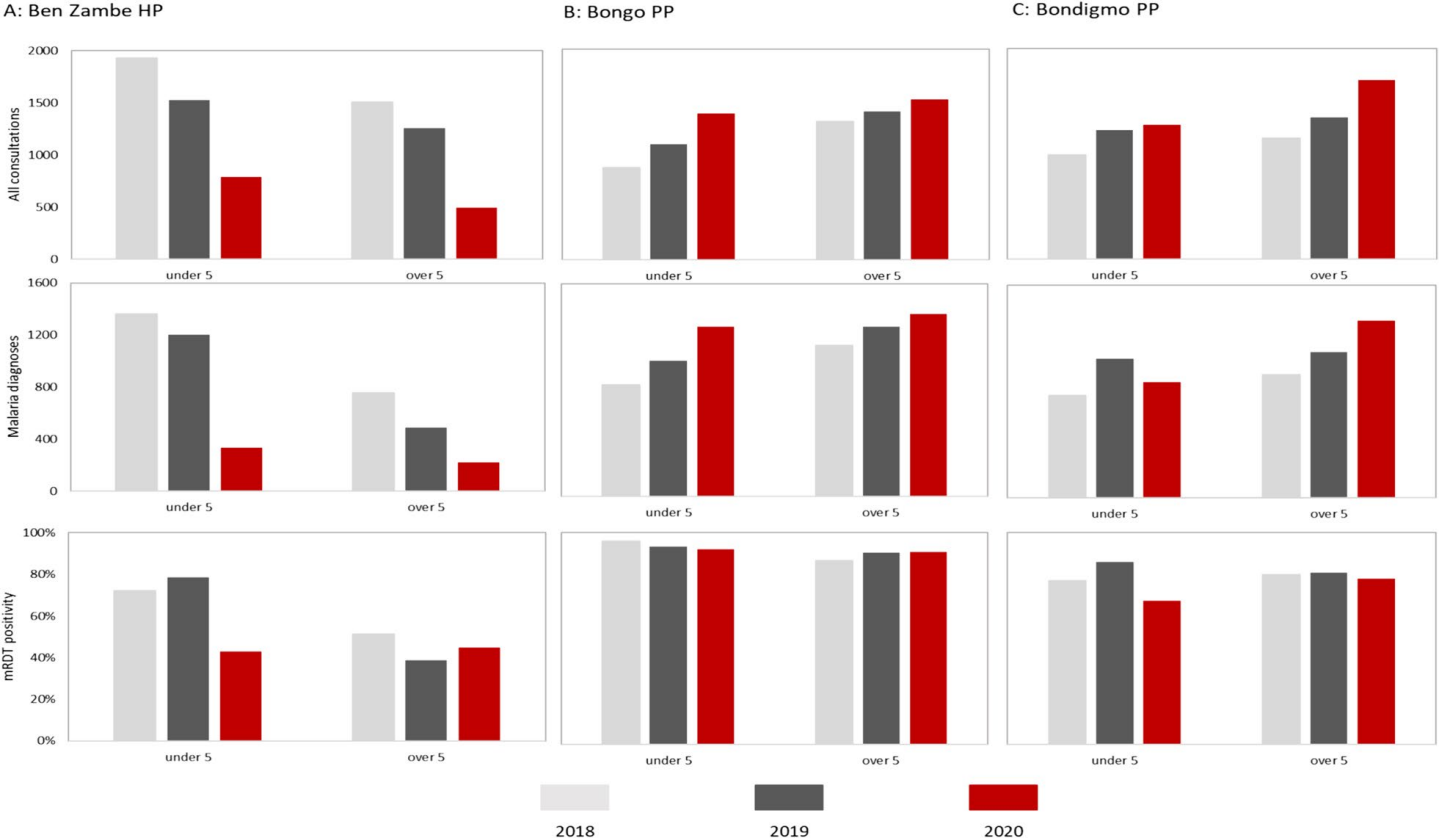
All consultations, malaria consultations, and proportion of total positive RDTs by age group, **a** Ben Zambé HP, **b** Bongo PP and **c** Bondigmo PP, weeks 37–48, 2018–2020

At Ben-Zambé HP, consultations and malaria diagnoses had been decreasing in the weeks prior to the MDA (Fig. 3A). From week 37, the first full week of the MDA in the commune of Ben-Zambé, the decreasing trend in consultations and malaria diagnoses became more marked in children under five years. Figure 4 shows the number of all consultation and the malaria consultations, and the RDT positivity in the three community MSF support HFs during the MDA period of 2020 and the same period of 2019 and 2018.

During the MDA period (weeks 37–48 2020), there was a 58.8% (95% CI 56.3–61.2%) decrease in all consultations and 70.6% (95% CI 67.9–73.1%) decrease in malaria diagnoses compared to previous years (Table 5). Statistically significant decreases were observed across both age groups. RDT positivity decreased by 42.9% (95% CI 36.0–49.0%) among those under 5 years, while it remained stable in those aged 5 years and older.

**Table 5:** Percentage reduction during the MDA period compared to the same period of 2018 and 2019 in the malaria indicators at MSF supported healthcare facilities in the Bossangoa project area by age group

Indicator	% Reduction in consultations, RDT positivity, hospital admissions and in-hospital deaths during the MDA compared to same period of 2018 and 2019		
	< 5 years	≥ 5 years	Total
Community HFs			
All consultations			
Ben-Zambé HP	**54.5 (50.8 to 57.9)**	**64.3 (60.3 to 67.6)**	**58.8 (56.3 to 61.2)**
Bondigmo PP	**− 15.1 (−23.3 to − 7.5)**	**− 36.3 (− 45.0 to − 28.2)**	**− 26.8 (− 32.7 to − 21.1)**
Bongo PP	**− 41.1 (− 51.2 to − 31.8)**	**− 11.6 (− 18.8 to − 4.9)**	**− 24.0 (− 29.9 to − 18.4)**
Malaria diagnoses			
Ben-Zambé HP	**73.7 (70.5 to 76.5)**	**64.3 (58.9 to 69.1)**	**70.6 (67.9 to 73.1)**
Bondigmo PP	4.2 (− 3.9 to 11.6)	**− 32.1 (− 41.6 to − 23.3)**	**− 14.9 (− 21.1 to − 9.1)**
Bongo PP	**− 37.3 (− 47.4 to − 27.9)**	**− 13.9 (− 21.8 to − 6.7)**	**− 24.1 (− 30.3 to − 18.3)**
% RDT positive			
Ben-Zambé HP	**42.9 (36.0 to 49.0)**	1.1 (− 14.1 to 14.3**)**	**29.4 (22.9 to 35.4)**
Bondigmo PP	**17.4 (10.4 to 23.8)**	− 3.1 (− 3.8 to 9.6**)**	**9.3 (4.4 to 14.0)**
Bongo PP	2.7 (− 4.5 to 9.4)	− 2.4 (9.4 to 4.2**)**	0.2 (− 5.2 to 4.5)
HRUB			
Admissions			
All admissions	**− 52.5 (− 81.1 to − 28.4)**	− 6.7 (− 95.7 to 41.9)	**− 48.3 (− 75.0 to − 25.7)**
Any malaria	**− 55.1 (− 88.1 to − 27.9)**	− 11.1 (− 240.7 to 48.7)	**− 52.0 (− 83.2 to − 26.0)**
Severe malaria	**25.1 (2.0–42.8)**	28.6 (− 98.3 to 74.3)	**25.4 (3.2 to 42.5)**
% Admissions due to severe malaria	**50.9 (35.8–63.5)**	33.0 (− 85.9 to 75.9)	**49.7 (34.7 to 61.2)**
In-hospital deaths			
All admissions			− 5.9 (− 237.5 to 47.2)
Any malaria			− 27.3 (− 328.8 to 50.7)
Severe malaria			75.0 (− 100 to 96.9)

A negative value indicates an increase. Values in bold have a p-value < 0.05

During the MDA there were statistically significant increases in consultations and malaria diagnoses overall and in those age over 5 five years at both Bondigmo and Bongo PPs (Fig. 4 and Table 3). At Bondigmo PP, the RDT positivity in those aged under 5 years did trend downwards during the early part of MDA period, with a decrease of 19.1% (95% CI 10.9–27.1%) during the MDA period compared to previous years. RDT positivity remained stable in those aged five years and over. At Bongo PP, the RDT positivity remained stable throughout the MDA period among both age groups.

#### Analysis of routine surveillance data-hospital admissions-results

**Fig. 5:**
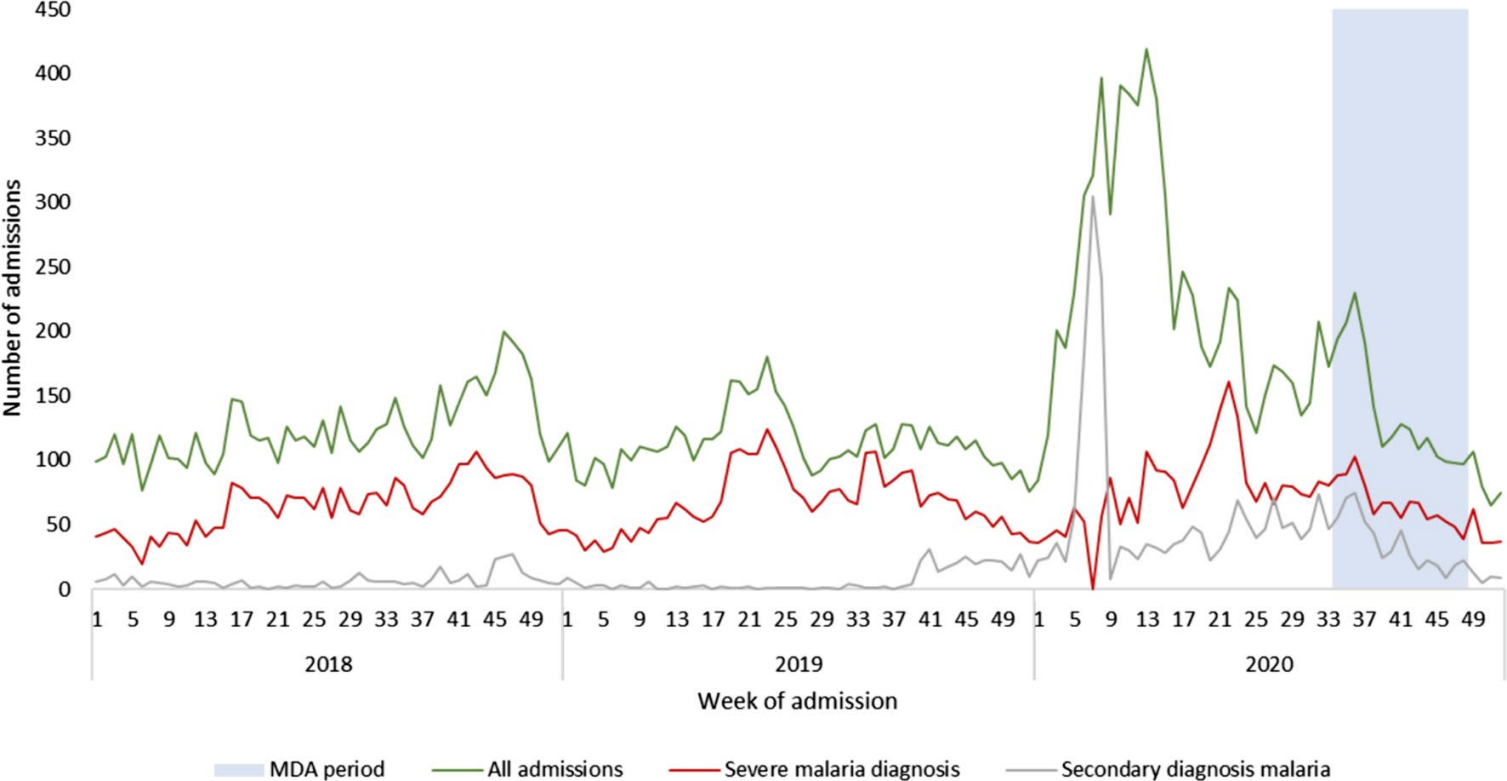
Weekly admissions of children up to 15 years of age to the paediatric unit of HRUB with a primary or secondary malaria diagnosis, 2018–2020

Up to the start of the MDA period in 2020, paediatric admissions to HRUB from all parts of the sub-prefecture were substantially elevated compared to previous years due to a large measles outbreak in early 2020 (Fig. 5). Admissions with a primary diagnosis of measles peaked in week 7 2020. All admissions remained elevated after the measles outbreak, likely related to both the direct and indirect effect of measles infection and possibly increased awareness of services at HRUB from outreach activities in the community as part of the outbreak response. Admissions from both MDA areas and non-MDA areas returned to normal levels in September 2020, at the time of the MDA.

During the MDA period (weeks 34–48) there was a statistically significant increase in all admissions and admissions with any malaria from the MDA area compared to previous years, while admissions with a primary diagnosis of severe malaria decreased by 25.4% (95% CI 3.2–42.5%). The number of in-hospital deaths among admissions from the MDA area was typically small over the period 2018–2020. There was not a statistically significant difference in the in-patient case fatality ratio during the MDA period among children from MDA intervention areas compared to previous years, nor between admissions from MDA intervention areas and non-MDA areas during the MDA period in 2020.

Given the small absolute numbers, a change in the annual number of deaths of only one or two can cause the case fatality ratio to vary considerably year on year, making comparisons difficult. Among children admitted from the MDA intervention area, there was also no statistically significant change in-hospital case fatality ratios during the MDA among children from the MDA intervention area compared with the same period in 2018 and 2019 (Table 4), nor between children admitted from the MDA intervention area and non-MDA areas in 2020 (Table 6).

**Table 6:** Case fatality ratios for in-hospital deaths among all admitted children up to 15 years of age, children admitted with any malaria diagnosis and children admitted with a primary diagnosis of severe malaria, from the MDA intervention area and non-MDA areas, HRUB, weeks 34–48, 2020

Indicator	MDA intervention area		Non-MDA areas	
	n/N	%	n/N	%
Deaths among all admitted children	9/244	3.7	66/1819	3.6
Deaths among children admitted with any malaria diagnosis	7/193	3.6	34/1327	2.6
Deaths among children admitted with a primary diagnosis of severe malaria	1/78	1.3	16/915	1.8

#### Potential adverse events

Among children who participated in round 3, 5.4% (n = 58/N = 1148; 95% CI 3.3–8.6%) of children were reported to have experienced illness in the week after administration. The most commonly reported symptoms were diarrhoea (n = 29; 47.4%; 95% CI 28.0–67.6%), nausea (n = 26; 45.0%; 95% CI 30.1–60.9%), rash (n = 6; 10.7%; 95% CI 3.1–30.7%) and headache (n = 4; 7.5%; 95% CI 2.4–32.2%). For 17.7% (95% CI 6.9–38.5%) of those who were reported to have experienced illness, the child was reported to have experience vomiting or nausea within one hour of administration of the MDA medication.

### Perceptions and experiences of community members

In total 388 people participated in the FGDs, of whom 65% were male (Table 7). Villages are labelled by a letter to avoid identification of the village. Similar themes emerged from both the questions on satisfaction with the MDA asked during the household survey and the FGDs, and therefore results of both will be presented together.

**Table 7:** Villages where FGDs were undertaken and the number of participants by sex, MDA evaluation study, Ouham, CAR, 2020

Round	Commune	Number of participants		
		Male	Female	Total
Round 1	Ouham-Bac	24	17	41
	Ndoromboli	35	16	51
	Ben Zambé	28	14	42
	Total	87	47	134
Round 2	Ouham-Bac	25	14	39
	Ndoro Mboli	29	10	39
	Ben Zambé	27	15	42
	Total	81	39	120
Round 3*	Ouham Bac	25	20	45
	Ndoro Mboli	25	13	38
	Ben Zambé	36	15	51
	Total	86	48	134
Total		254	134	388

The improved health of the children and reduction in malaria was the most prominent theme to emerge:

*"In my household also, the children keep getting sick and it's always malaria, I keep bringing them to the hospital but after the distribution of the antimalarial drugs …things are better now"* [FGD round 3, Village S, Ouham-Bac]

Participants also spoke of fewer emergency healthcare visits and less deaths related to malaria:

*“Before the drugs arrived, many children lost their lives to malaria…There has been a change in the village and the children are doing well. Before, we go back and forth between Village V and Gbadé and sometimes we lose the children along the way…”* [FGD round 3, Village V, Ndoro-Mboli]

The improved health of the children also meant capacity of the households to attend to other tasks. They were freer to attend to farm work, as they were not caring for sick children:

*"…we liked it because we can now go to work in our fields and do our business without worrying”* [FGD round 2, village J, Ouham-Bac]

The fact that the medicine was distributed in the village was appreciated as it did not incur transport costs, and did not take up too much time for the household so they could continue with their daily activities after the distribution:

*“…I bring my child to the fixed site and then go home and wander to these daily occupations”* [FGD round 3, village Z, Ben-Zambé]

Participants spoke of how the MDA brought a sense of peace, because previously the financial barriers to healthcare where a major burden on families:

*“…if these drugs were not distributed, some parents who cannot afford to buy the drugs to treat their children will do what? Many children in the village are dying of malaria just because the parents don't have the money to take them to the hospital”* [FGD-round 2, Village M, Ndoro-Mboli]

FGD participants reported that initially there was hesitancy amongst some of the community members due to a fear of side effects from the MDA drugs. There were also some rumours about the MDA including that it was trying to infect their children with COVID-19 and that the medicine was a new drug manufactured in Europe that MSF wanted to test. However, participants also revealed that those who did not participate in round 1 due to such suspicions, did participate in subsequent rounds after seeing that the children did not suffer negative consequences and were in good health:

*“In the first round of the administration… some parents were fleeing following false information circulating…and think that these drugs could cause problems for their children. But, during the 2nd round, they find that the children who were administered in the 1st round are in better shape than those who are not administered and ended up registering their children in the 2nd round”* [FGD round 3, Village S, Ouham-Bac]

One village reported that the distribution day in round 2 clashed with market day which led to reduced participation:

*“The mothers of children had all gone to sell in the market…the weekly market …is the only day when we can sell and have some money to meet our needs in the area"* [FGD round 2, Village M, Ndoro-Mboli]

The importance of community engagement and the role community leaders was emphasized during the FGDs. Participants appreciated the formal meetings held in the villages and the formal communication through official letters with the village chief as it showed respect for the community leaders. They also appreciated the involvement of the community leaders and local authorities in the recruitment of ReCos. The participants attributed the success of the MDA to the promotion by community leaders:

*“For me, what I saw, the village chief himself cried out in the evening for all the mothers to come together and stay at home to wait for the medicines. The chief asked each mother to tell her neighbour and to sensitize the others who were in the fields, to bring the children back to drink the medicines…It was the village chiefs who encouraged the women to participate in the distribution. Our spiritual fathers also sensitized us during worship and their wives did so during women’s religious meetings”* [FGD round 1, Village G, Ben-Zambé]

They also recognized the contribution of all the community as a whole:

*"We all contributed to this administration without knowing it, I give a concrete example, it happens sometimes that some mothers of children take their children to go to the field, but the other woman gives advice to her neighbour not to go to the field but to accompany the children to be administered”* [FGD round 3, Village G, Ouham-Bac]

However, when it was perceived that a community was not involved, or another village was given preferential treatment this caused tension:

*“We have in this village two chefs but…you based yourself on only one…please do your best to remedy this difficulty which can create a dispute between us”* [FGD round 3, Village AA, Ben-Zambé]

Participants wanted the MDA to continue. The main suggestion was that the MDA be expanded to include more people. While participants were very grateful for the MDA and how it improved the health of their children, they were concerned about their own health and that they too needed to be healthy to care for the children:

*“What about us moms? You must also give us so that we are healthy and have the strength to feed them”* [FGD round 1, Village H, Ben-Zambé]

Others advocated for younger infants and pregnant women to be included.

## Discussion

MSF delivered three rounds of MDA, a total of 134,117 courses to over 40,000 children across three communes of Bossangoa sub-prefecture in 2020.

### Coverage and acceptability of the MDA

Acceptance of the MDA was high as demonstrated by the high coverage achieved-estimated at > 95% for each round by the household survey and at over 70% or over 85% per round using the MDA administrative data, depending on the population estimate used. Inaccurate population estimates due to a lack of a recent census are a limitation of each coverage estimate and ongoing population movement likely contribute to the discrepancy between methods. Despite the limitations of the three coverage estimates, all approach or exceed the WHO recommendation that coverage of > 80% is required for an MDA to be successful [3]. This high coverage is consistent with the satisfaction with the MDA expressed by community members during the FGDs and through the household survey. The community members asked for the MDA to be repeated and to expand the eligibility. FGDs on malaria prevention and control in the Bossangoa area in 2019 indicated that the community would accept an MDA so the high community acceptance is not surprising [16]. The FGDs conducted during the evaluation did reveal initial hesitancy and even suspicions towards to the MDA, which were subsequently addressed in health promotion activities conducted as part of the MDA. The COVID-19 pandemic may have added to an environment favourable to suspicions. However, in other MDAs elsewhere before COVID-19, suspicions and rumours also circulated in the community [17]. The WHO guidelines on MDA implementation recommend plans to detect and address rumours are included in MDA planning such as building relationships with local media to mitigate against them spreading false information [4, 8].

The main reasons for non-participation identified through the household survey were primarily logistical due to absences such as working in the fields or foraging. FGD participants highlighting that clashes between the MDA distribution and market day prevented their participation in the MDA also serves as a reminder to consider the competing priorities for the community members in improving coverage of community interventions.

The WHO recommended a coverage of > 80% of the target population for an MDA [4]. Community engagement has been recognized as vital for a successful MDA [4, 18]. The MSF MDA team did undertake substantial community engagement activities during the planning phases and throughout the MDA. Informal and formal meetings were held with community leaders to discuss and the concept and goal of an MDA campaign during which the community’s assent to proceed and to participate were achieved. The community were also directly involved in the delivery of the MDA through the employment of local ReCos [19]. The MDA team included anthropologists who accompanied the team when visiting villages for training and administration. They provided valuable support in understanding of context and population, as well as the effects of the MDA on communities and providing real time feedback to improve and adapt implementation of the MDA (see Additional file 4 for details of community engagement during the MDA). During the FGDs, the community members did recognize the role their leaders and they themselves played in the success of the MDA.

### Clinical impact on malaria morbidity and mortality

Estimates from several evaluation components indicate that the MDA in Bossangoa did reduce morbidity, particularly among children five years and under. The household survey found that morbidity in the preceding four weeks was higher in children who had not participated in the final MDA round. The enhanced surveillance data collected during the MDA, showed that the odds of having a positive RDT was higher among does presenting to Ben-Zambé HP who had not participated in the MDA in the preceding four weeks compared to those who had. Also, in Ben-Zambé HP, there was a substantial reduction of 59% and 71% in the number of all consultations and malaria diagnoses, respectively during the MDA period compared to the same period of preceding years, while RDT positivity among those aged under five years was 43% lower. At HRUB, while hospital admissions increased by 53% during the MDA period, admissions with a principal diagnosis of severe malaria decreased by 25%. However, routine surveillance data did not demonstrate a consistent effect across all HFs, which complicates the assessment of the clinical impact. Increases in consultations during the MDA period were seen in the two PPs. The reason for some of the increases during the MDA period is not clear. There was no known population movement in the area to explain the increase. The health promotion associated with the MDA may have increased awareness of the PPs and the free care available. This may have had a particular impact on the PPs as they are in more remote locations towards the outer edges of the commune, and MSF teams had not previously visited the surrounding villages. At HRUB, admissions had been elevated prior to the MDA, since a large measles outbreak in early 2020, and only began to return to normal levels around the time of the MDA which may explain the increase in admissions.

This evaluation, which yielded mixed findings, including some suggesting an impact on morbidity and mortality within the target group, contributes to the sparse existing literature on MDAs in emergency contexts. Most prior studies have demonstrated an effect, although with some ambiguity: a 2021 Cochrane review concluded that, based on results from a single trial, MDA probably reduces parasitaemia incidence, but does not reduce parasitaemia prevalence at one to three months after MDA in moderate to high‐transmission settings [20]. Observational studies on the real-world effectiveness of MDAs and other chemoprevention programmes in reducing malaria illness in humanitarian crises are scarce [21–23]. MSF implemented an MDA with 2 rounds of artesunate-amodiaquine (ASAQ) and 1 round of 3-day regimen of artesunate-pyronaridine in the Ituri region of the Democratic Republic of Congo (DRC) from September 2020 to January 2021. Before and after retrospective mortality studies across MDA and non-MDA areas were undertaken. They found that in MDA areas the under-five mortality rate (U5MR) decreased from 2.32 (95% CI 1.48–3.16) deaths/10,000 population before the MDA to 1.10 (95% CI 0.5–1.71) deaths after the MDA [21]. In comparison, in areas where the MDA had not been implemented, the U5MR was stable during the same periods. Additionally, the U5MR and malaria-specific mortality was significantly higher in non-MDA areas after the MDA, with adjusted rate ratios of 2.17 (95% CI 1.36–3.49) and 2.60 (95% CI 1.56–4.33), respectively. In 2015, an intermittent preventive treatment of malaria in children (IPTc) programme consisting of 3 rounds of DHA-PQ at 8-week intervals was implemented by MSF in two refugee camps in northern Uganda among children aged 6 months to 14 years. Compared to the preceding year, the incidence of malaria decreased with an incidence rate ratio (IRR) of malaria of 0.73 (95% CI 0.69–0.77) among children under 5 years of age; and 0.70 (95% CI 0.67–0.72) among children aged 5–14 years [22].

Several MDA programmes were implemented during the 2014–2016 Ebola outbreak in West Africa. In Liberia, MSF undertook two rounds of MDA with ASAQ in Monrovia. Household surveys conducted after the distribution found that the incidence of self-reported fever decreased from 4.2% in the month prior to the first round of MDA to 1.5% after round one (p < 0.001) [23]. In Sierra Leone, the Government and its partners implemented an MDA with two rounds of ASAQ at 5-week intervals during December 2014–January 2015 [24]. Segmented time-series analysis applied to weekly data from 49 primary health units and 11 hospitals during the study period, found that malaria RDT positive cases decreased by 47% (41–52%) at week 1 post-first and remained lower throughout all post-MDA weeks; the RDT test positivity rate declined by 35% (32–38%) at week 2 and stayed low throughout all post-MDA weeks. In addition to differences in the MDA protocols in terms of medicines, rounds and intervals, the use of different methods and indicators to evaluate the clinical impact makes it difficult to compare results with this MDA in Bossangoa sub-prefecture.

One of the valuable contributions of this evaluation to existing literature is the addition of qualitative data collection capturing the perspectives of the community on the impact of MDAs. The most striking indicator of a clinical impact from the MDA in Bossangoa came from the qualitative components of the evaluation, where the perceived positive impact by community members was clear and substantial. During both the FGDs and the household survey the community members reported that the children were no longer sick and visits to HFs had decreased. The community members also reported an indirect positive impact of the MDA on social wellbeing of the family and community. They described a reduction in the opportunity costs that arise when a child is sick, such as having to stay home from the fields to care for the child. They also reported that healthcare expenditure had decreased. Similar descriptions of these indirect benefits were not found in the literature.

Notably, during the household survey no deaths among children were identified. If the same crude mortality rate as found in recent retrospective mortality studies in CAR [7, 25], approximately 8 to 11 deaths would have been expected to have been identified among the sampled children. As fever/malaria accounted for 30% of deaths in those aged under 5 years in that other study, the MDA should have decreased the expected number of deaths substantially. The sample size of the household survey may not have been adequate to detect them.

It should also be noted that the MDA commenced later than initially planned and toward the end of the typical seasonal rise of malaria cases. This was partly due to a large measles outbreak and COVID-19 preparedness activities hindering planning. A clearer impact on morbidity and mortality may have been achieved if the MDA commenced earlier.

Of note, the feared impact of COVID-19 in CAR was not observed during 2020 and there was no evidence of widespread COVID-19 related illness in the MDA area during the intervention of the evaluation. As of 17 August 2020, the start of the MDA, 4679 COVID-19 confirmed cases and 61 deaths had been reported across CAR [26]. By the end of 2020, 4970 cases had been reported. No case had been detected in the MDA communes during the MDA. The first confirmed case was not detected at Bossangoa hospital until 2021 [27]. While the number confirmed cases is very likely to be underestimated, due to many reasons including access to care and testing, MSF facilities in the area did not observe an increase in consultations or admissions with respiratory disease during 2020.

### Future malaria control activities

The cornerstone of malaria control and prevention in humanitarian emergencies remain timely diagnosis and treatment of malaria infections [8]. There is also good evidence for the effectiveness of ITNs and some data that shows good protection by indoor residual spraying (IRS) in humanitarian emergencies [8]. MDAs might be a complimentary addition to the toolbox of malaria control interventions in emergencies. The evaluation was able to show a high acceptability and coverage despite extremely challenging logistics in a complex emergency including conflict and the COVID-19 pandemic simultaneously. There was some evidence previously for the effectiveness of MDAs in emergency settings and this evaluation added to this, showing a reduction of malaria morbidity, severe disease, and mortality in children under 5. However, it did not show the reduction uniformly across all data sources and did not show a reduction in older age-groups (despite targeting individuals up to 15 years).

A more standardized methodology for evaluating malaria prevention interventions, such as LLIN, IRS, IPTi or packages of interventions in humanitarian emergencies, including common epidemiological endpoints, would help inform the selection and prioritization of interventions [28, 29]. In the interim, while further evidence and information amasses, decision-makers will need to base decisions on other contextual factors such as past and present intervention coverage; acceptability; equality and equity of access and use; and available resources while ensuring the core intervention of LLIN and case management achieve high coverage. In CAR, both access to diagnosis and treatment, as well as bed net use, indicate room for improvement [7, 16]. Better access to care, increased bed net distributions, tailored health promotion community engagement, and the combination of MDAs with activities to distribute bed nets or IRS could provide options for future malaria prevention and control strategies in CAR. Whichever the strategy of choice, proper evaluation will be key to learn more about best ways to reduce the burden of malaria amongst conflict affected populations.

### Limitations

The evaluation has several limitations. The estimate of coverage through the household survey relied principally on verbal reports which potentially over-estimated participation. Confirmation of participation by card would for all cases have been preferable. Households reported that the MDA cards were kept by the ReCo, or that the hut they were in was locked as household members were in the fields and therefore could not be accessed to show the survey team. In one cluster, the households had hidden all their documents including the MDA cards in the bush due to the increasing tensions in the area and fears their houses may be pillaged by armed groups, a reminder of the volatility of the setting.

That 19% of households were absent on the study day may have led to an over estimation of the coverage. For the most part, neighbours reported that absent households were in the fields undertaking agricultural activities. These households may have been more likely to have been absent during the MDA also and therefore not participated. Children being away from the village due to working in the fields was noted during the MDA itself.

A social desirability bias may have been present during the household survey and FGDs, with false reports of participation or a positive experience, in particular if participants perceived that a positive response would influence future MDAs or the provision of other MSF supported services in the area.

The analysis of routine surveillance data is limited by the fact that it only includes three MSF supported community HFs, all of which were in one commune. The MDA was originally planned for an area with a greater MSF presence but due to increased insecurity this was changed. The analysis of routine surveillance data may also be limited due to the usual data quality issues seen with surveillance data such as coding practices. However, there is no reason to believe this would have changed during the MDA period to prevent comparisons with previous years.

Finally, the association between non-participation in round 3 of the MDA and recent illness, may be confounded by other factors. For example, poorer families may have had increased risk of illness due to worse living conditions and also may have been more likely to have been absent during the MDA due to field work.

### Strengths

A major strength of this study is the use of mixed methods and triangulation of results from different components. The use of qualitative components in particular the FGDs to ascertain the community member’s perceptions of the MDA provided insights on wider impact such as a decrease in healthcare spending which would not have emerged through quantitative evaluations alone.

## Conclusions

It was feasible, albeit challenging, for MSF to deliver an MDA in CAR. This was thanks to the determination and motivation of the MDA team. The required change in intervention area due to conflict, and the delays due to the measles outbreak response and COVID-19 preparedness activities do show that flexibility and agility are required given the precarious nature of CAR. The evaluation showed very high levels of coverage and of community acceptance and satisfaction. The extent of community involvement was likely a contributing factor to the MDA’s success.

There was some evidence of a reduction in morbidity, particularly in those under five years of age. However, this reduction was not seen across all indicators explored. A change in in-hospital mortality among admissions aged under 15 years was not observed. Nevertheless, community members emphasized that the MDA had a positive impact on children’s health, and also reported other indirect benefits which enhanced family and community wellbeing.

The cornerstone of malaria control in prevention in humanitarian emergencies remain timely diagnosis and treatment of malaria infections [7]. Efforts to ensure good access to these, in addition to strategies with good evidence for the effectiveness such as ITNs should be prioritized. MDAs and other chemoprevention interventions may be an additional tool of value if contextual factors support it. Given the resource intensive nature of this MDA, further exploration of alternative designs in terms of the target group or delivery to reduce mortality among the most vulnerable should be considered. A standardized approach to evaluation of malaria interventions will enable comparison in terms of impact and other contextual factors and aid decisions makers to develop effective and sustainable strategies in these challenging settings.

### Supplementary Information

Additional file 1.

Additional file 2.

Additional file 3.

Additional file 4.

## Data Availability

The datasets used and/or analysed during the current study are available from the corresponding author on reasonable request.

## Abbreviations

AE: Adverse event
ANC: Antenatal care
ASAQ: Artesunate-amodiaquine
CAR: Central African Republic
95% CI: 95% Confidence interval
DEFF: Design effect
DHA-PQ: Dihydroartemisinin-piperaquine
DRC: Democratic Republic of the Congo
FGD: Focus group discussion
HC: Health centre
HF: Health facility
HIS: Health information system
HP: Health post
HRUB: *Hôpital régional universitaire* (Regional university hospital) Bossangoa
ICASEES: *Institut Centrafricain des Statistiques et des Etudes Economiques et Sociales* (Central African Institute of Statistics and Economic and Social Studie*s)*
IPTc: Intermittent preventive treatment of malaria of children
IPTi: Intermittent preventive treatment of malaria of infants
IPTp: Intermittent preventive treatment of malaria in pregnancy
IPTsc: Intermittent preventive treatment of malaria of school children
IRS: Indoor residual spraying
ITN: Insecticide treated net
MDA: Mass drug administration
RDT: Malaria rapid diagnostic test
MSF: Médecins sans Frontières
PMC: Perennial malaria chemoprevention
PP: *Point palu* (Malaria post)
ReCo: *Relais communautaire* (Community health worker)
SMC: Seasonal malaria chemoprevention
TFC: Therapeutic feeding centre
U5MR: Under 5 mortality rate
WHO: World Health Organization
