# Supplementary material for: Mixed-method evaluation study of a targeted mass drug administration of long-acting anti-malarials among children aged 3 months to 15 years in the Bossangoa sub-prefecture, Ouham, Central African Republic, during the COVID-19 pandemic
Source: Malar J. 2024 May 15;23:146. doi: 10.1186/s12936-024-04968-1 (PMC11094902; doi:10.1186/s12936-024-04968-1)
Supplement: Supplementary file 2 — Additional file 2. [file 12936_2024_4968_MOESM2_ESM.docx]

### Additional file 2: Topic guide used for focus group discussions

### Ref: Submission ID bee6e371-23b4-495f-8283-ff004e162588

*Mixed-method evaluation study of a targeted Mass Drug Administration of long-acting antimalarials among children aged 3 months to 15 years in the Bossangoa sub-prefecture, Ouham, Central African Republic, during the COVID-19 pandemic*

| **MSF – OCA: PROGRAMME GROUPES DES DISCUSSION de l’AMM, 2020** | | | | | | | | | |
| --- | --- | --- | --- | --- | --- | --- | --- | --- | --- |
| **Equipe: ____________________________**__________________________________ | | | | | | | | | |
| **Date (*jj/mm/aaa*:** | _____ / _____ / _____ | | | | | | | **Village :** | _________________ |
| **Leiu du groupe de discussion:** | | | __________________________________________________________ | | | | | | |
| **Membres du group de discussions:** | 🞏  🞏  🞏 | Parents/gardiens  Leaders communautaires  Agents de santé communautaire | | | |  |  | | |
| **Nombre total de personnes participant au groupe de discussion:** | | | | | | | | _________________________________ | |
| **Langue du groupe de discussion:** | | | | ___________________________________ | | | | | |
| **Introduction:** Avant de commencer le groupe de discussion, présentez-vous à partir de MSF. Expliquez que MSF est là pour savoir ce que la communauté a pensé du récent programme de distribution de médicaments aux enfants pour prévenir le paludisme dans la région. Expliquez que nous voulons savoir comment se sent la commanuté afin de savoir comment l'améliorer ou la changer à l'avenir. Demandez-leur de lire ou lisez-leur la fiche d'information. Posez-leur les questions dans le consentement verbal de. Si vous avez leur consentement, continuez.  **But du groupe de discussion :**  Nous voulons comprendre l'expérience des gens de la récente intervention de l’AMM | | | | | | | | | |
| **Thème 1: Prévention du paludisme** | | | | | | | | | |
| ***Question générale*** | | | | | ***Invites potentielles*** | | | | |
| *Nous savons que le paludisme est un problème de santé qui affecte les communautés ici; nous parler des moyens de l'éviter?* | | | | | - Dites-nous ce qui est le plus efficace pour vous? Pourquoi? | | | | |
|  | | | | | | | | | |
| **Thème 2: Perspectives générales sur l’AMM** | | | | | | | | | |
| ***Question générale*** | | | | | ***Invites potentielles*** | | | | |
| [Si non mentionné dans la question précédente]  *Au cours du dernier [calendrier], MSF et le Ministère de la Santé et de la Population ont mené une AMM [très brève explication]; Dites-nous ce que vous pensez de ça?* | | | | | - Qu'aimez-vous / la communauté à ce sujet? - Quelles sont vos inquiétudes? - Comment pensez-vous que cela fonctionne? - Combien de temps pensez-vous que les enfants seront protégés après avoir pris le médicament? - Pensez-vous que vous devez encore utiliser d'autres moyens de prévenir le paludisme lorsque vous prenez les médicaments? | | | | |
| **Theme 3: Participation à l’AMM** | | | | | | | | | |
| ***Broad question*** | | | | | ***Invites potentielles*** | | | | |
| *Dites-nous comment les gens d'ici ont réagi à l’AMM, la plupart des gens y ont-ils participé ou non? Pourquoi?* | | | | | - Pourquoi les gens ont-ils choisi de participer? - Pourquoi les gens ont-ils choisi de ne pas participer? - Qu'est-ce qui encouragerait plus de gens à participer à l'avenir? | | | | |
| **Theme 4: Expériences de l’AMM** | | | | | | | | | |
| **Question générale** | | | | | ***Invites potentielles*** | | | | |
| *Quelles ont été les expériences de participation à l’AMM?* | | | | | - Que pensez-vous de la façon dont le médicament a été distribué? Qu'est-ce que vous avez aimé / détesté? (Avez-vous dû attendre longtemps?) - Que pensez-vous de la visite du ReCo chaque jour pour voir l'enfant prendre le médicament? - Les enfants ont-ils eu des problèmes après avoir pris les médicaments? Si oui, parlez-nous d'eux? - Les enfants ont-ils pris les médicaments pendant 3 jours? Si oui, pourquoi? Sinon, pourquoi pas? - Pensez-vous que le temps, les jours et la saison de l'administration étaient appropriés? - Si le programme recommençait l'année prochaine, y participeriez-vous? - Que pensez-vous des tranches d'âge auxquelles les médicaments ont été administrés? | | | | |
| **Thème 5: Information et engagement sur l’AMM** | | | | | | | | | |
| ***Question générale*** | | | | | ***Invites potentielles*** | | | | |
| *Racontez-nous comment vous avez entendu de l’AMM?* | | | | | - Pensez-vous avoir reçu suffisamment d'informations sur le programme? - Comment la communauté a-t-elle été impliquée dans l’AMM? Comment pensez-vous que cela pourrait être amélioré? - Comment pensez-vous que le village peut aider dans l’AMM? | | | | |
| **Suggestions et autres** | | | | | | | | | |
| - Dites-nous comment vous pensez que l’AMM pourrait être amélioré? - Dites-nous autre chose que vous aimeriez partager dont nous n'avons pas déjà discuté? - Des questions pour nous? | | | | | | | | | |
