# Supplementary material for: Mixed-method evaluation study of a targeted mass drug administration of long-acting anti-malarials among children aged 3 months to 15 years in the Bossangoa sub-prefecture, Ouham, Central African Republic, during the COVID-19 pandemic
Source: Malar J. 2024 May 15;23:146. doi: 10.1186/s12936-024-04968-1 (PMC11094902; doi:10.1186/s12936-024-04968-1)
Supplement: Supplementary file 3 — Additional file 3. [file 12936_2024_4968_MOESM3_ESM.docx]

### Additional file 3: Comparison of symptoms between children that participated in round 3 of the MDA and those that did not.

### Ref: Submission ID bee6e371-23b4-495f-8283-ff004e162588

Mixed-method evaluation study of a targeted Mass Drug Administration of long-acting antimalarials among children aged 3 months to 15 years in the Bossangoa sub-prefecture, Ouham, Central African Republic, during the COVID-19 pandemic

Symptoms reported among children who were reported to have been ill during the preceding 4 weeks by participation in round 3 of the MDA, MDA coverage survey, Ouham, 2020.

| **Symptom/illness** | **Total** | | **Ill and did participate in R3** | | **Ill and did not participate in R3** | | **p- value** |
| --- | --- | --- | --- | --- | --- | --- | --- |
|  | **n** | **% (95% CI)** | **n** | **% (95% CI)** | **n** | **% (95% CI)** |  |
| Gastrointestinal | 24 | 2.0 (1.1—3.7) | 17 | 1.5 (0.7—3.0) | 7 | 12.0 (0.7—19.2) | <0.001 |
| Respiratory | 8 | 0.7 (0.3—1.5) | 2 | 0.2 (0.00—0.8) | 6 | 8.5 (1.9—30.5) | <0.001 |
| Fever | 6 | 0.5 (0.3—1.2) | 4 | 0.4 (0.2—1.1) | 2 | 2.8 (0.5—14.7) | 0.04 |
| Headache | 4 | 0.4 (0.0—2.1) | 1 | 0.0 (0.0—0.7) | 3 | 6.1 (2.0—17.2) | <0.001 |
| Rash | 3 | 0.2 (0.0—0.9) | 2 | 0.2 (0.00—0.7) | 1 | 1.3 (0.0—10.6) | 0.01 |
| Musculoskeletal/  arthralgia | 2 | 0.2 (0.0—1.6) | 0 |  | 2 | 4.1 (1.3—11.7) | <0.001 |
| Conjunctivitis | 1 | 0.0 (0.0—0.6) | 1 | 0.0 (0.0—0.7) | 0 |  | 0.83 |
| Malaria symptoms not further specified | 1 | 0.0 (0.0—0.6) | 1 | 0.0 (0.0—0.7) |  |  | 0.90 |
| Malnutrition symptoms not not further specified | 1 | 0.0 (0.0—0.6) | 1 | 0.0 (0.0—0.7) |  |  | 0.83 |
| Shivers |  | 0.1 (0.0—0.8) | 0 |  | 1 | 2.0 (0.7—6.0) | <0.001 |
