# Supplementary material for: Mixed-method evaluation study of a targeted mass drug administration of long-acting anti-malarials among children aged 3 months to 15 years in the Bossangoa sub-prefecture, Ouham, Central African Republic, during the COVID-19 pandemic
Source: Malar J. 2024 May 15;23:146. doi: 10.1186/s12936-024-04968-1 (PMC11094902; doi:10.1186/s12936-024-04968-1)
Supplement: Supplementary file 4 — Additional file 4. [file 12936_2024_4968_MOESM4_ESM.docx]

### Additional file 4: Community engagement activities

### Ref: Submission ID bee6e371-23b4-495f-8283-ff004e162588

### *Mixed-method evaluation study of a targeted Mass Drug Administration of long-acting antimalarials among children aged 3 months to 15 years in the Bossangoa sub-prefecture, Ouham, Central African Republic, during the COVID-19 pandemic*

The MDA team engaged and involved the community in the MDA through several mechanisms during the planning, administration and evaluation phases of the MDA.

- Initial informal meetings with community leaders
  - Community engagement commenced as the MSF team were doing initial explorations of the area to check road conditions and security etc. When the team arrived at villages for the first time they met with community leaders present. Subjects that were discussed included: MSF (principles, identity, activities globally and locally), follow-up on the recent measles vaccination campaign, community morbidity and mortality in the last three months, health facility structures in the vicinity and their services as well as reference/transfer capabilities, COVID-19 information spread by the MOH, other salient community issues.
- Formal meetings with community leaders
  - The next step in community engagement consisted of formal meetings between the MSF team and key community leaders. These included chef de groupement/village/quartier, chef of the health post/centre, pastors, school directors, traditional midwifes, responsible person of women / young men groups, etc. Community members received invitations via a letter sent to the chef de village to attend these meetings. Meetings lasted 1-2 hours, at a location proposed by the chef. Soda and biscuits were provided to all participants. Points discussed during the meeting included:
    - MSF (principles, identity, activities globally and locally)
    - MDA campaign (concept and goal, and if community thinks it could help – ownership/participation)
    - Number of ReCo’s needed per village.
    - Questions and answers
- Employment of ReCos from the community
  - ReCos were selected from the community, often working in their own village. They attended training with the MSF team covering the MDA concepts, methods of administration, their role etc. The ReCo’s used the two days ahead of the first administration day to promote the location, time and day, and importance of participation to the campaign in their villages.
- Inclusion of social scientists in the MDA team
  - The MDA team included three anthropologists. They provided valuable support in understanding of context and population, as well as the effects of the MDA on communities. During the training and administration phases the distribution team was accompanied by the anthropology team. The anthropology team collected field notes on the perspectives and feedback from communities. These were used to support implementation and provide ‘real time’ feedback to improve and adapt implementation. The anthropologist also undertook the FGDs reported on in the manuscript. Provisional results from the focus group discussions were provided to the MDA team lead to improve the programme where relevant.
